# Immobilized pH gradient-driven paper-based IEF: a new method for fractionating complex peptide mixtures before MS analysis

**DOI:** 10.1186/1559-0275-8-10

**Published:** 2011-06-15

**Authors:** Beerelli Seshi, Kumaraguru Raja, KH Chandramouli

**Affiliations:** 1 Department of Pathology and Laboratory Medicine, Los Angeles Biomedical Research Institute at Harbor-UCLA Medical Center, 1124 West Carson Street, Torrance, California 90502, USA; 2 Current address: 580 Cross Point Parkway, Getzville, NY 14221, USA; 3 Current address: Section of Marine Ecology and Biotechnology, Division of Life Science, The Hong Kong University of Science and Technology, Kowloon, Hong Kong SAR

**Keywords:** Mass spectrometry, iTRAQ, Offgel electrophoresis, Paper IEF, Progenitor cells, Clinical proteomics

## Abstract

**Introduction:**

The vast difference in the abundance of different proteins in biological samples limits the determination of the complete proteome of a cell type, requiring fractionation of proteins and peptides before MS analysis.

**Methods:**

We present a method consisting of electrophoresis of complex mixtures of peptides using a strip of filter paper cut into 20 sections laid end to end over a 24-cm-long IPG strip, the pH gradient of which would drive the electrophoresis. Peptides absorbed onto individual paper pads after electrophoresis are subsequently recovered into a buffer solution, thus dividing a complex peptide mixture according to pI into 20 liquid fractions. This paper-based IEF method (PIEF) was compared side-by-side with a similar but liquid-based Offgel electrophoresis (OGE) by analyzing iTRAQ-labeled peptide mixtures of membrane proteins from four different cell types.

**Results:**

PIEF outperformed OGE in resolving acidic peptides, whereas OGE did a better job in recovering relatively basic peptides. OGE and PIEF were quite comparable in their coverage, identifying almost equal number of distinct proteins (PIEF =1174; OGE = 1080). Interestingly, however, only 675 were identified by both of them, each method identifying many unique proteins (PIEF = 499; OGE = 415). Thus, the two methods uncovered almost 40% more proteins compared to what is obtained by only one method. **Conclusion**: This initial investigation demonstrates the technical feasibility of PIEF for complementing OGE. PIEF uses standard IPG IEF equipment, requires no specialized apparatus (e.g., OGE fractionator) and may be integrated into peptide mapping strategies for clinical samples.

## Introduction

The complexity of the human proteome, in terms of its size (over 100,000 proteins/variants) and dynamic range (up to a billion-fold difference in abundance of the various types of proteins), is well recognized [1,2]. Because MS methods preferentially identify the most abundant proteins in complex mixtures [3], the ability to identify low-abundance proteins by MS requires application of a variety of pre-MS techniques for depleting and/or separating out abundant proteins [4-12] as well as techniques for fractionating peptides [13-17]. Of these techniques, Offgel electrophoresis (OGE), with the capability to resolve proteins as well as peptides by IPG IEF with subsequent liquid-phase recovery [17], is proving quite powerful in providing greatly improved protein coverage [18,19]. Because peptide IPG IEF is compatible with iTRAQ [20], OGE is finding valuable applications in quantitative proteomics as well [21,22]. However, OGE requires the use of a relatively specialized OGE fractionator. Here we report the development of a similar IPG gel-driven, paper-based IEF method (PIEF) that is equally powerful in fractionating peptides but does not require specialized equipment. We tested the utility of PIEF by employing iTRAQ-labeled peptide mixtures and a side-by-side comparison with OGE both in terms of peptide recovery and proteomic coverage.

## Results

### Evaluating the Efficacy of PIEF

**Figure 1:**
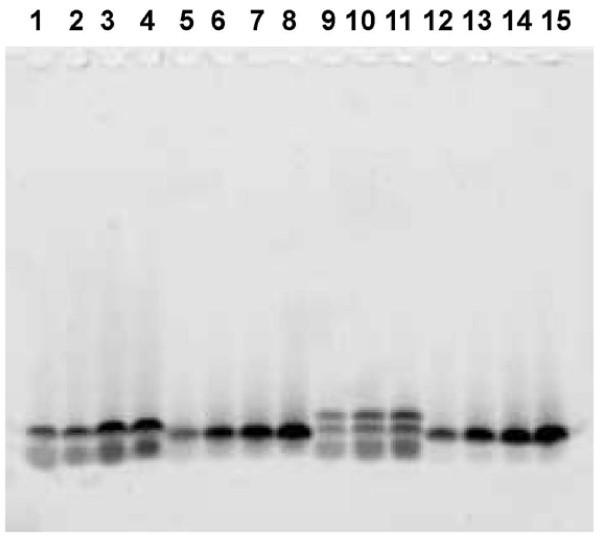
**Standardization of a mini SDS-PAGE gel system capable of resolving Cy3-labeled known and synthetic peptides, loaded in increasing amounts**. Lanes 1-4: angiotensin. Lanes 5-8: bradykinin. Lanes 9-11: synthetic peptide 72109. Lanes 12-15: synthetic peptide 72120.

**Figure 2:**
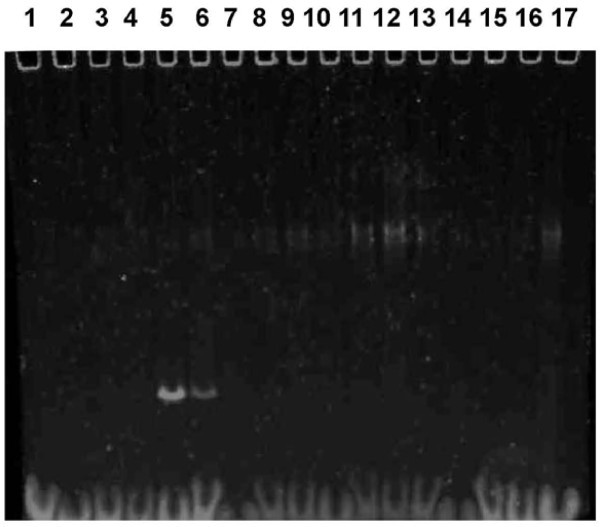
**Mini gel SDS-PAGE analysis of protein fractions resulting from PIEF using a known protein, beta lactoglobulin, and a pH 3-10 IPG strip (gel stained with Sypro Ruby)**. Lanes 1-17: fractions from paper strips 1-17.

**Figure 3:**
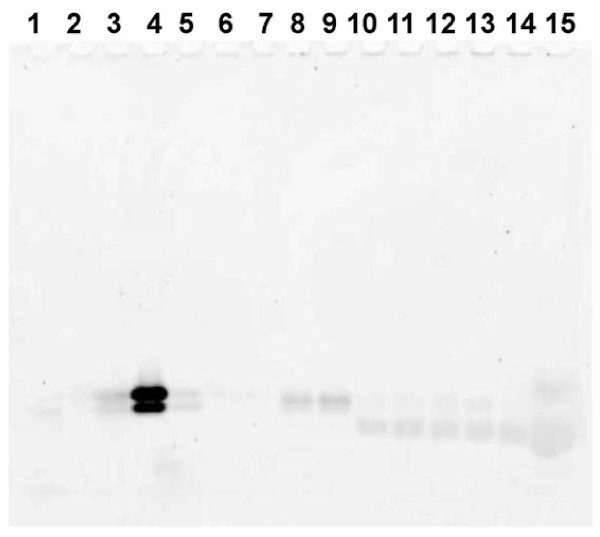
**Mini gel SDS-PAGE analysis of peptide fractions resulting from PIEF using a Cy3-labeled model synthetic peptide 72109 on a pH 3-10 IPG strip**. Lane 1: left electrode pad fraction 1. Lane 2: left electrode pad fraction 2. Lanes 3-14: fractions from paper strips 1-12. Lane 15: sample buffer.

We first investigated conditions for setting up a simple gel system that could resolve small peptides with the objective of monitoring IEF fractions of a peptide sample. As shown in Figure 1, the gel adequately resolved different naturally occurring as well as synthetic peptides. The utility of PIEF was first tested using a known small protein, beta lactoglobulin (BLG), because BLG is routinely used for testing OGE (Offgel Fractionator Kit Quick Start Guide). BLG predictably focused into two paper strips (filter pads) on the acidic end of the pI 3-10 IPG strip, corresponding to its two known isoelectric species on this strip (Figure 2). PIEF was next tested using a synthetic peptide 72109. This peptide essentially focused into one filter pad on the acidic end of the IPG strip, correlating with its theoretical pI of 4.4 (Figure 3). Thus, PIEF yielded the expected results for BLG and for the peptide 72109 in terms of peptide migration and resolution. The selected peptide *standards *necessarily have known pI values. Although they do not stain well with Sypro Ruby, they could be labeled with Cy3, and be used to track whether they would actually migrate to the expected pI locations as determined by the underlying IPG strip. Thus we were able to optimize the PIEF buffer composition and running conditions and establish PIEF's basic functionality.

The full utility of PIEF was then tested by using iTRAQ-labeled peptide mixtures of *cytosolic proteins *from four different cell samples, identifying and quantifying the relative levels of 1,053 *non-redundant *proteins (minimal set of proteins excluding duplicate identifications) at the >95% confidence level (data not shown). PIEF was finally tested side-by-side with OGE by analyzing iTRAQ-labeled peptide mixtures of *membrane proteins *from the four cell types, as described below.

### Comparing the Performance of PIEF and OGE

**Figure 4:**
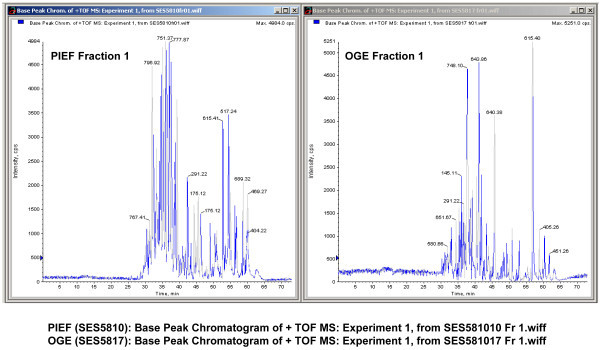
**Base peak chromatograms of PIEF vs. OGE presented side-by-side showing excellent and comparable signal-to-noise ratios**. Fraction 1 of PIEF vs. Fraction 1 of OGE.

**Figure 5:**
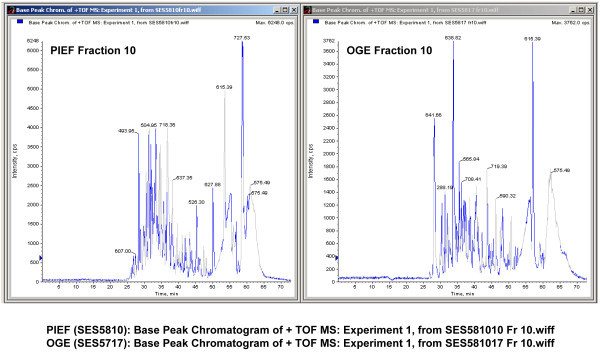
**Base peak chromatograms of PIEF vs. OGE, Fraction 10 of PIEF vs. Fraction 10 of OGE**.

**Figure 6:**
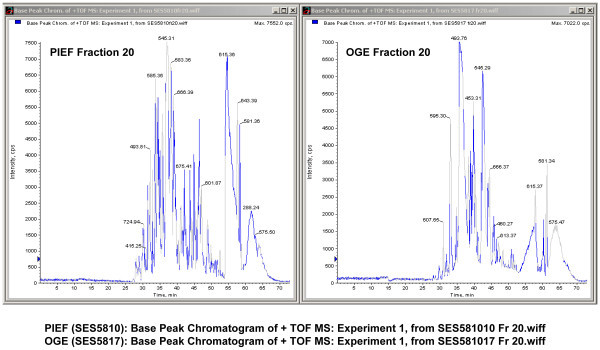
**Base peak chromatograms of PIEF vs. OGE, Fraction 20 of PIEF vs. Fraction 20 of OGE**.

Base peak chromatograms as presented in Figure 4, Figure 5 and Figure 6 show excellent and comparable signal to noise ratios. The fact that PIEF has identified even greater number of proteins and with greater level of confidence than OGE, using the same instruments and identical running and analysis settings, suggests that low signal to noise ratio is not an issue with the use of PIEF (see under section "*At the protein level"*).

### At the peptide level

**Figure 7:**
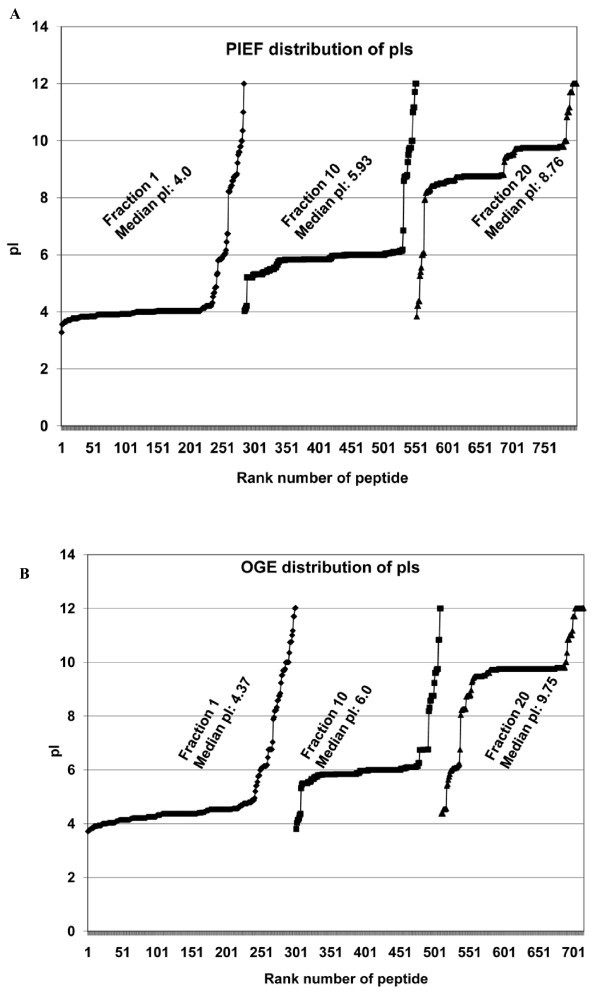
**Comparative distribution of peptide pIs along the pH gradient in PIEF vs. OGE (plots A and B, respectively)**. The *x *axis represents the rank number of peptide as the peptides were first sorted in ascending order of pI value before plotting, and the *y *axis represents the pI. The *x *axis appears thick because each peptide is represented by a small vertical bar (>700 such bars are juxtaposed along the axis).

**Figure 8:**
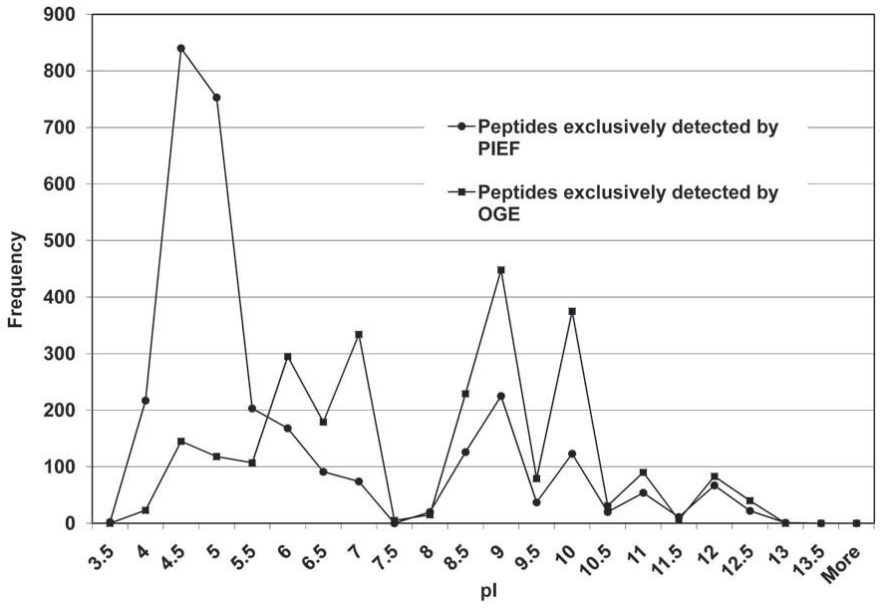
**Comparative distribution of pIs of peptides detected exclusively by PIEF or OGE (3054 and 2602 peptides, respectively)**.

PIEF overall identified 9, 812 peptides, of which 4,951 were non-redundant, whereas OGE overall identified 8,141 peptides, of which 4,499 were non-redundant. As expected, both methods recovered acidic peptides from the acidic end (fraction 1), basic peptides from the basic end (fraction 20), and peptides with intermediate pIs from the middle of the gel (fraction 10) (Figure 7A and 7B). The pIs of the peptides recovered from each filter pad overall were consistent with the pI range of the underlying part of the IPG strip, demonstrating successful fractionation of a complex mixture of peptides generally according to pI. Combining the peptide lists from both methods led to identification of 7,553 non-redundant peptides. Of these, 3,054 peptides were exclusively detected by PIEF, the majority (~60%) appearing within the pI range 4.0-5.5 (Figure 8), whereas 2,602 peptides were exclusively detected by OGE, the majority (~60%) appearing within pI ranges 6.0-7.0 and 8.5-10.5 (Figure 8); 1,897 peptides were common to both methods (not shown). Thus, PIEF outperformed OGE with respect to recovery of acidic peptides, whereas OGE did a better job in resolving relatively basic peptides.

### At the protein level

PIEF identified 1,174 non-redundant proteins, and OGE identified 1,090 non-redundant proteins at the >95% confidence level, resulting in a combined 1,589 non-redundant proteins, with corresponding iTRAQ ratios for all of them. Although the difference in the total number of proteins identified by these methods was only 84, there were 499 proteins exclusively identified by PIEF and 415 proteins exclusively identified by OGE. A total of 680 proteins were identified by both the methods. However, ratios were available for all cell-types for 675 of them, and only these have been considered in further analysis.

We next investigated whether the 675 proteins identified by both methods yielded iTRAQ ratios that were comparable between methods. Because it is a common practice to use correlation coefficients for comparing two proteomic or transcriptomic methods, we determined the r-values between PIEF vs. OGE for iTRAQ ratios of these 675 proteins. The results were as follows: PIEF Log HSF6: MPC 117:114 vs. OGE Log HSF6: MPC 117:114, *r = 0.8987*; PIEF Log HSF6 : SFC 117:116 vs. OGE Log HSF6 : SFC 117:116, *r = 0.9116*; PIEF Log MPC : SFC 114:116 vs. OGE Log MPC : SFC 114:116, *r = 0.9100*.

**Figure 9:**
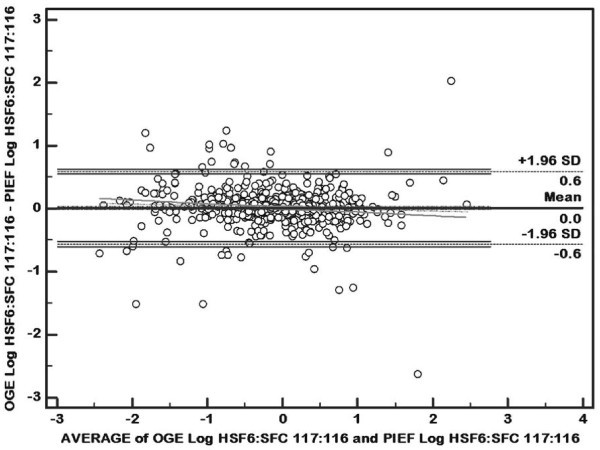
**Bland and Altman (mean vs. difference) plots showing the level of agreement of iTRAQ ratios for 675 proteins identified by both PIEF and OGE**.

Although the correlation coefficients between PIEF vs. OGE methods were satisfactory, they may not be reliable indicators of agreement between different methods, and thus the use of r-values for method comparison is practically prohibited in clinical sciences, such as clinical chemistry, as highlighted by Bland and Altman [23]. To determine the extent of agreement/disagreement between the results for PIEF and OGE, we further analyzed our data via mean vs. difference plots of Bland and Altman (for example, see Figure 9 for 117:116). Similar plots were obtained for the other ratios. The standard deviations of the differences were very similar in magnitude (the equality of the two means being expected due to the normalization procedure of the ProteinPilot software). The Bland-Altman plot also shows that there was no systematic dependence of the difference on the average value, indicating reasonable agreement between the two methods over the entire range of ratios. The r-values have been included because it is a pervasive practice. In view of the possible limitations of r values as discussed by Bland and Altman, we wanted to demonstrate the robustness of our results by presenting additional evidence using Bland-Altman plot.

The most striking aspect of this comparison, however, is seen when one looks at the list of unique proteins**--**i.e., identified by only one of the two methods. This result is especially important from the perspective of uncovering expanded proteomic coverage. Supplementary data files list the genes encoding proteins identified by both methods (Additional file 1, Table S1), exclusively by PIEF (Additional file 2, Table S2), or exclusively by OGE (Additional file 3, Table S3). The Supplementary Tables include the MS data and the associated information, such as, Accession Number, Gene Symbol, Gene Description, N (Rank of the specified protein), Unused (ProtScore), Total (ProtScore) and %Cov. *The MS data are provided for each method and differentiated for the proteins detected in common and for those detected by one method only.*

IPI recognizes protein isoforms as separate entities using unique protein accession numbers. Evaluation of Tables S2 and S3 in terms of IPI Accession Numbers vs. Gene Symbols revealed that, of the 914 proteins that were exclusively identified by either method, namely 499 for PIEF and 415 for OGE, 129 were protein isoforms whereas 784 were products of distinct genes; i.e., 86% of the total were distinct gene products. In any case, the purpose of application of the Bland and Altman plots is to assist in the comparison of different assays, thereby defining an optimum assay(s) for a particular analysis.

Table 1 summarizes the results from Supplementary Data tables (Tables S1-S3). The results show, a) PIEF consistently outperformed OGE by not only identifying a greater number of proteins but also by increased level of confidence with which they are identified, and b) the proteins identified by both methods are identified with a higher level of confidence by both methods in comparison to proteins identified by one method alone. Overall, our data demonstrate that PIEF and OGE cannot substitute for one other; rather, the methods are complementary.

**Table 1:** Summary of average MS data scores for proteins identified by OGE and PIEF

Number of Proteins Identified	%Cov (OGE)	%Cov (PIEF)	Total (OGE)	Total (PIEF)	Unused (OGE)	Unused (PIEF)
**Proteins Common to PIEF and OGE (675) (Table S1)**	20.73	23.64	9.28	10.70	8.78	10.13

**Proteins Unique to PIEF (499) (Table S2)**	N/A	14.66	N/A	4.79	N/A	4.54

**Proteins Unique to OGE (415) (Table S3)**	14.09	N/A	4.58	N/A	4.38	N/A

N/A: not applicable

See Supplementary Data Tables S1-S3 for lists of individual proteins with the associated MS data, from which the above average-scores were obtained. The MS scores reflect on the confidence with which the proteins are identified. See Results for an interpretation of the findings.

### Accuracy of iTRAQ Ratios

**Figure 10:**
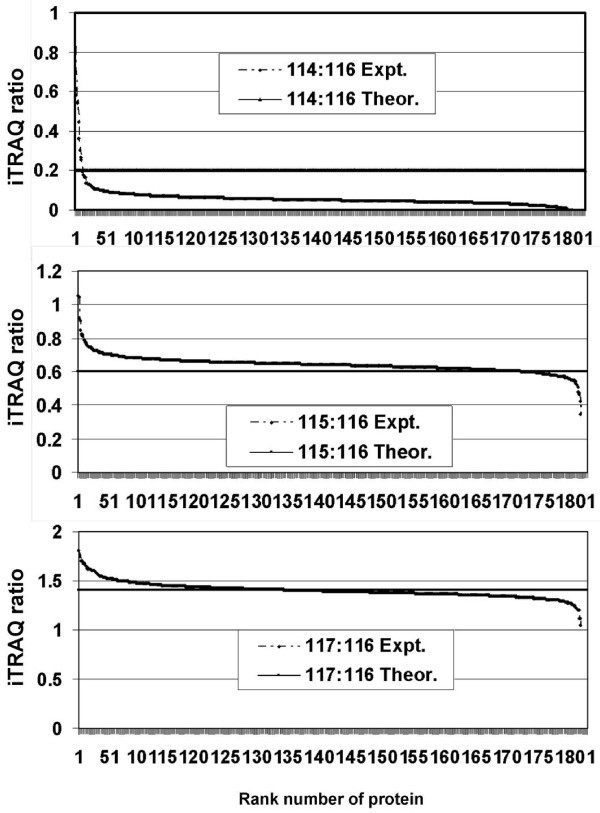
**Controlled quantitation study to test the accuracy of iTRAQ ratios**. The *x *axis represents the rank number of protein as the proteins were first sorted in descending order of iTRAQ ratio before plotting, and the *y *axis represents the iTRAQ ratio. The *x *axis appears thick because each protein is represented by a small vertical bar (>800 such bars are juxtaposed along the axis).

Known amounts of a particular complex protein mixture were differentially labeled, multiplexed, and processed through all the steps of OGE prior to MS analysis. In Figure 10, the proteins identified (801 proteins out of perhaps thousands present in the sample) are shown on the *x *axis, and the iTRAQ ratios are on the *y *axis. The observed mean ratio vs. expected ratio was 0.051 vs. 0.200 (in case of iTRAQ label 114), 0.641 vs. 0.600 (in case of 115), and 1.402 vs. 1.400 (in case of 117). The experimentally observed ratios were systematically lower when the theoretical ratios were low (i.e., 0.2, based on the amounts added; Figure 10, top panel). However, comparing the three panels in Figure 10 actually shows that increasing the amount of protein in the numerator (keeping the amount of protein in the denominator constant) increased the agreement between the expected and experimental ratios. Thus, the higher the abundance of protein measured (in absolute amount) in samples being compared, the greater the agreement between the expected and the experimental ratios (Figure 10). We did not perform this analysis using PIEF due to limitations of laboratory resources.

## Discussion

### Protein-level Fractionation Alone is Insufficient for Complete Proteomic Analysis

Two critical factors limit the identification of low-abundance proteins in proteomic analyses: differences in protein abundance, and the yield of diagnostic peptide fragments during MS analysis. A variety of methods exist for fractionation of proteins before MS analysis to facilitate detection of low-abundance proteins (for review, see [7,12]). These methods include separation based on cellular organelles (e.g., cytosolic, plasma membrane, nuclear, and cytoskeletal fractions), affinity purification, and biochemical properties like hydrophobicity, MW, pI, and high-resolution 2-D PAGE that combines MW and pI [24], although 2-D PAGE is not amenable to shotgun proteomics. Yet, these methods are not sufficient for in-depth proteomic analyses because the complexity generated following tryptic digestion of proteins is completely independent of the level of complexity that existed at the protein level. As reported earlier [25] for example, 11% (80 of 712) of proteins in the MPC proteome generated 50% of the peptides (5,258 of 10,506) that were detected during LC-MS/MS of the MPC proteome. This peptide-rich fraction of the MPC proteome would potentially hinder detection of low-abundance and small proteins, because the latter necessarily generates a small fraction of the peptides in the total MPC proteome. Higher coverage of the proteome may be achieved by combining fractionation both at the protein and peptide levels. In the present study, proteins were first fractionated based on subcellular location, followed by fractionation of peptides based on pI, which indeed greatly enhanced the proteomic coverage in comparison to the previous study [25].

### Methods of Peptide Fractionation

Traditionally, 2-D LC-MS/MS analysis of peptides entailed SCX followed by reversed-phase chromatography [26]. Because of limited resolution of SCX, IPG IEF was developed in place of SCX [15]. However, IPG-IEF requires excision of small sections of the IPG strip followed by extraction of peptides in the gel matrix. Consequently, a more elegant IPG gel-driven but liquid-based OGE was described [17]. However, OGE requires a specialized apparatus--the OGE fractionator. Here we describe PIEF that uses standard equipment. However, PIEF and OGE surprisingly provided very different results when applied to iTRAQ-labeled membrane protein samples (see below). This suggests that either method alone is insufficient and rather that they are complementary. In any event, these methodologies will certainly require further investigation because OGE thus far has stood alone without being challenged, except for a single comparative study that actually found reversed-phase HPLC at high pH to be superior to OGE [27].

### Possible Reasons for Disparate Results between PIEF and OGE

PIEF and OGE outputted significantly different results in several respects. Each method recovered large numbers of unique peptides; PIEF did a better job at recovering/identifying acidic peptides, whereas OGE identified a greater number of basic peptides. In fact, for many of the proteins that were identified by both methods, the magnitudes of the corresponding iTRAQ ratios seemed to be significantly different. The possible reasons for the disagreement between the results by PIEF and OGE may be several-fold. Neither PIEF nor OGE may be optimal because substantial amounts of sample peptides were left behind in the IPG gel matrix, as assessed by imaging of Cy3-labeled peptides remaining in the IPG strips after either analysis (results not shown). To our knowledge, OGE has not been investigated from this perspective. We do not know whether there was differential retention of different peptides because we did not quantify the peptides that were retained in the gel matrix. Differential retention and/or non-uniform extraction could potentially impact the iTRAQ ratios. These issues remain to be investigated.

Four additional reasons for differences between PIEF and OGE are discussed here. These reasons were unavoidable as it can be realized from the following discussion. First, we had to use different IEF/extraction media, 4M urea/10% glycerol/0.5% ampholytes for Offgel as recommended by the manufacturer, and 7M urea/2M thiourea/1%DTT/1% ampholytes for PIEF. Because the Offgel IEF medium was found to be suboptimal for PIEF during initial trials, we used the latter medium for PIEF. Such wide chemical difference may well explain some differences in the performance of OGE vs. PIEF. Second, the gaps between pads in PIEF and the gaps between wells in OGE may not necessarily correspond with each other, and the peptides migrating to those locations are essentially lost to analysis. Imaging of the post-run IPG strip after completion of IEF using Cy-labeled peptides showed bright bands representing these junctions (data not shown). PIEF experiments using one long uncut paper strip spanning the length of the IPG strip, or using small pads (as was done here) but leaving no gaps in between them, were unsuccessful due to poor fractionation (data not shown). Because it would be cumbersome to cut a long strip of wet paper after IEF, we first tried the method with 20 pads and it proved to be the best approach in terms of resolution. We believe that one long strip of paper spanning the entire length of IPG gel somehow interferes with the underlying electrophoresis, or that peptides once they are absorbed onto the filter paper transform the paper into a parallel conducting medium; the absence of gaps compromises resolution. When gaps are present, however, once peptides have migrated to their pI point and are absorbed to the corresponding pad they are probably trapped. The decision to use 20 pads was based on cost and the labor required on one hand and the extent of desired fractionation on the other, because the better the resolution the greater the proteomic coverage. Because each of the 20 fractions represents a separate MS study with associated costs, we thought 20 fractions would be experimentally manageable. Third, the efficiencies of peptide extraction by PIEF and OGE, even from the functional locations of the gel (i.e., the gel areas corresponding to pads in the case of PIEF and to wells in the case of OGE), may not be high as alluded to above, and consequently the data may not necessarily be comparable. Finally, for PIEF the peptides were mechanically extracted by vortexing the paper pads, whereas for OGE the peptides were allowed to spontaneously diffuse into the IEF running buffer. These issues may not apply to reversed-phase HPLC at high pH or conventional SCX chromatography, but of course SCX yields relatively poor resolution and consequently lower coverage.

### Accuracy and Precision Issues Inherent to iTRAQ Quantitation

The overall differences in the results outputted by PIEF vs. OGE were probably compounded by the variability inherent to iTRAQ quantitation. A number of reports have addressed the issues of iTRAQ quantitation accuracy (defined as being close to the true value and measured by statistical mean) and precision (defined as reproducibility and measured by standard deviation) [28-32]. The variability in iTRAQ ratios can be potentially related to various peptide properties that include abundance, length, sequence, mass, etc. More specifically, it has been reported that iTRAQ is sensitive to errors in precursor ion selection in comparison to ICAT and DIGE, suggesting mass spectrometer-related interference could introduce substantial random variation [32]. Our own observations suggest that the agreement between the expected and the experimental ratios correlates with the abundance of protein measured (in terms of absolute quantity) in samples being compared (Figure 10), suggesting that protein abundance is fundamental to the reliability of the relative quantitation. In any event, the primary focus of the present investigation was on maximizing the coverage. To this end, we attempted to characterize as many different subproteomes as possible, rather than making replicate measurements on the same sample; this approach is consistent with the practice reported in most studies of this type. We were able to identify and quantify relative amounts of ~4,750 non-redundant proteins by studying 200 subproteomes (10 fractions at the protein level, with each protein fraction further fractionated into 20 peptide-level subproteomes) across four different cell types (results to be published). These results are in contrast to those from our earlier study in which there was no fractionation at the protein level, and SCX fractionation was used at the peptide level; that study identified only 900 proteins [25].

### Potential Applications of PIEF

The primary purpose of developing PIEF was to decrease the complexity of proteomic samples by fractionating each individual sample at the peptide level, thereby enhancing the coverage of the proteins identified. Because PIEF uses standard IPG IEF equipment and thus requires no specialized equipment like the OGE fractionator, it may be routinely integrated into standard proteomic workflow, thus lending PIEF to widespread applications, including peptide-mapping studies involving human body fluids [33].

The peptide SDS-PAGE system provided a direct and relatively inexpensive method to monitor the initial performance of PIEF as well as OGE before MS analysis of complex mixtures of peptides. Although Sypro Ruby staining of the gels was not particularly helpful for detection of peptides (probably because of its lower sensitivity), Cy3 labeling of peptides proved to be powerful (Figures 1 and 3). Following Cy3 labeling, a particular peptide migrates to its pI region and is absorbed onto the paper pad, whereas the free dye migrates to the basic end of the gel and is absorbed onto the paper pad placed over the right end of the gel. This finding suggests a means to prepare labeled peptides free of unbound dye molecules.

PIEF could potentially be used for preparative-scale purification of isoelectrically equivalent forms of peptides employing first a broad pH-range IPG strip (e.g., pH 3-10) and then perhaps a narrow pH-range strip as relevant to the peptide(s) of interest. The success of the method could be monitored by peptide SDS-PAGE, as described above.

In conclusion, a substantial number of proteins were detected by OGE but missed by PIEF, and vice versa. Pooling data from several such complementary fractionation methods is likely to provide a much better experimental estimate of the proteome size.

## Methods

All studies were conducted according to the Institutional Review Board policies and practices of the Los Angeles Biomedical Research Institute.

### Cell Culture

Normal bone marrow samples were obtained from healthy volunteers at the Hematopoietic Stem Cell Facility at Case Western Reserve University Comprehensive Cancer Center. Human marrow stromal mesenchymal stem cells (MPC) were culture-expanded using a marrow aspirate sample from a 25-year-old normal white male, and MPC were purified using a Percoll gradient as described [34]. Human embryonic stem cells HSF6 (XX, 46, NIH no. UC06) were maintained on irradiated CF1 murine embryonic fibroblasts in Knockout Dulbecco's modified Eagle Medium supplemented with 20% knockout serum replacer (Invitrogen, Carlsbad, CA), fibroblast growth factor-2 (4 ng mL^-1^), 1 mM [L-glutamine, 0.1 mM nonessential amino acids, and 0.1 mM 2-mercaptoethanol with daily medium changes, and passaged every 4-6 days by incubation with 1 mg mL^-1 ^dispase/collagenase IV (Invitrogen) for 12-18 min at 37°C. Adult human dermal fibroblasts were obtained commercially (Lonza, Inc, Walkersville, MD) and were expanded according to the manufacturer's instructions using fibroblast cell basal medium supplemented with 2% fetal bovine serum, 0.1% insulin, 0.1% gentamycin and amphotericin, and 0.1% fibroblast growth factor-beta.

### Protein Fractionation and Subsequent Sample Cleanup

Each of the four cell samples, namely, purified MPC, unpurified stromal cells (USC), HSF6 (H6C), and skin fibroblast cells (SFC), was fractionated into cytosolic, membrane, nuclear, and cytoskeletal protein fractions, referred to as q-proteome fraction 1 (QF1), QF2, QF3 and QF4, respectively, using the Q-Proteome Cell Compartment kit (Qiagen Inc, Valencia, CA) and following the manufacturer's instructions with additional protease inhibitors. The fractionated proteins were acetone-precipitated using four volumes of cold acetone, and incubated for 2 h at -20°C and overnight at 4°C [35]. Protein precipitates were collected by centrifugation at 21920 × *g *at 4°C for 30 min using 2-mL tubes in a Microfuge 22R Centrifuge (Beckman Coulter, Brea, CA) or at 22000 × *g *at 4°C for 30 min using 15-mL tubes in a Avanti J25 (Beckman Coulter) and air-dried for 15-30 min. Protein pellets were stored at -80°C. Before use, protein precipitates were resolubilized and proteins were reduced with tributylphosphine (Sigma, St. Louis, MO) and alkylated with acrylamide using a reduction/alkylation kit (Proteome Systems, Woburn, MA). Protein concentrations were estimated using an assay that avoids interference from reducing agents and detergents (***r***educing agent ***c***ompatible and ***d***etergent ***c***ompatible (RC DC) Protein Assay, Bio-Rad, Hercules, CA). A measured amount of a reduced/alkylated protein sample in 1 mL was desalted by performing three rounds of centrifugation at 4000 × *g *for 60 min at 25°C, using Amicon Ultra-4 Centrifugal Filter Units (Millipore, Billerica, MA), an Allegra 25 R centrifuge (Beckman Coulter) with 15-mL swinging buckets, each time diluting the sample in 3-4 mL deionized solution of urea (8.75 M)/thiourea (2.5 M) and generating a retentate volume of 250-500 µL. After final centrifugation, the sample proteins were acetone-precipitated into 800 µg or 110 µg aliquots and stored at -80°C.

We have been aware that the precipitate of membrane proteins with organic solvents would not re-dissolve readily. As outlined above, we circumvented this problem by pre-aliquoting the sample proteins in the amount (110 μg) as required for subsequent labeling with a given iTRAQ reagent and then precipitating the protein sample using acetone. As described below, the precipitate was re-dissolved using Invitrosol (LC/MS Protein Solubilizer, a surfactant blend, Invitrogen, Inc.) in triethylammonium bicarbonate buffer, followed by trypsin digestion. Even if one were to assume dissolution was incomplete, trypsin will digest the protein anyway. The completion of digestion was routinely tested by SDS-PAGE gel.

### Tryptic Digestion of Proteins, iTRAQ Labeling and Multiplexing of Peptides, and Subsequent Sample Cleanup

Aliquots of acetone-precipitated samples (110 µg assuming ~10 µg loss during these processes) were resuspended in 40 µL of 5× Invitrosol without ammonium bicarbonate, 40 µL of 125 mM triethylammonium bicarbonate buffer, pH 8.5 and 120 µL of H_2_O. Samples were vortexed for 1-2 min and incubated at 60°C for 5 min, followed by vortexing for 1-2 min. They were then incubated at 60°C for 10 min, followed by sonication for 30 min. Proteins were digested using 5 µg of trypsin per 100 µg of sample protein and incubating at 37°C overnight. Digestion was terminated by adding TFA (0.5% final concentration) to the samples. The peptides were vacuum-dried and stored frozen at -80°C. iTRAQ labeling of the peptide samples was performed using the iTRAQ Reagents Multiplexing kit [36] according to the manufacturer's instructions (Applied Biosystems, Inc., Foster City, CA). The samples were labeled as MPC-114, USC-115, SFC-116, and H6C-117 (depending on the iTRAQ reagent used) and were multiplexed and vacuum dried. Desalting of the vacuum-dried peptide samples was carried out using OASIS extraction cartridges (Waters, Milford, MA). Briefly, the peptide sample was resolubilized in 1 mL of 0.1% TFA and passed through a column conditioned with 1 mL of 100% ACN and equilibrated with 1 mL of 0.1% TFA. The bound peptides were first washed with 4 mL of 0.1% TFA followed by 800 µl of H_2_O, and eluted using two rounds of 250 µl of 70% ACN. Initially, cytosolic protein samples (QF1's) were used to test the utility of PIEF. Next, membrane proteins (QF2's) were used to compare PIEF with OGE. For the purpose of comparison, the eluted peptides *at this stage were equally divided*, keeping the technical variables constant thus far. The peptides were then dried and resolubilized in IEF sample buffer, followed by application to PIEF and OGE.

### Fractionation of iTRAQ-labeled Peptides using PIEF

PIEF was performed using the IPGPhor system and 24-cm IPG strips, pH 3-10 (GE Healthcare, Piscataway, NJ). Notably, an IPGphor strip holder with cup loading (GE Healthcare) was used instead of regular strip holders, although no cup loading of the samples was employed. The IEF strip was rehydrated in a rehydration tray overnight at room temperature (RT) in 450 µl of an IEF buffer (7 M urea/2 M thiourea/1% DTT/1% 3-10 ampholytes) with gel face down and overlaid with 1.5 mL of dry strip cover fluid (GE Healthcare). Each rehydrated strip was transferred to a strip holder with the gel side facing up, as is done for a cup-loading setup, so that paper pads could be placed in direct contact with the gel surface. Strips of filter paper (GE healthcare 18-1004-40) were cleanly cut in advance into 10 mm-long IEF electrode strip pads using a paper cutter (FISKARS). The strip pads were then wetted on a glass plate with 2 drops of IEF buffer for each pad using a 1-ml pipette, and they were finally laid end to end on top of the IPG strip, with the first pad beginning at the + sign of the strip and leaving a ~1-mm space between the pads. The strip pads were not necessarily from one strip, but from two or more strips of the same lot number. Sample solution prepared using 450 µl IEF buffer was loaded in the central 10 pads by adding 40-45 µl of sample on top of each pad until the entire sample was loaded. Then 40-45 µl of IEF buffer (without sample peptides) was added to the remaining peripherally located pads. Pads were gently pushed against the gel. Two 5 mm-long IEF electrode pads were wetted with deionized H_2_O, followed by blotting the excess water with Kim Wipes and then placing one pad at each end of the IPG strip. The electrodes were positioned at each end such that they were on top of the electrode pad with IPG gel underneath. Dry strip cover fluid (3 ml) was added on top of the pads. IEF was run under the conditions as outlined in Table 2.

**Table 2:** Running conditions for IEF Phase I

S1	Step and hold	500 V	0.5 kV-h
S2	Gradient	1000 V	1.0 kV-h
S3	Gradient	8000 V	13.5kV-h
S4	Step and hold	8000 V	60kV-h
S5	Step and hold	500 V	20 h

At the day's end when the run reached Step S4, the electrode pads were replaced with fresh ones (5 mm long) wetted with deionized H_2_O and blotted almost completely dry on KimWipes. If necessary, 1.5 ml of dry strip cover fluid was added and the run was restarted by creating a new program with conditions as shown in Table 3, changing Step S1 according to the stage when S4 was stopped such that the run entails a total 63kV-h including both phases.

**Table 3:** Running conditions for IEF Phase II

S1	Step and hold	8000 V	23 kV-h
S2	Step and hold	500 V	20 h

After the run, each of the 20 paper pads was transferred to a 2-ml tube containing 200 µl of extraction buffer (7 M urea, 2 M thiourea) and were vortexed vigorously for 30 s followed by centrifugation at 21920 × *g *at 20ºC for 5 min using a Microfuge 22R centrifuge (Beckman Coulter). The solution was recovered by pressing the pad using a pipette tip. The extraction was repeated (x2), and the extracted samples from each pad were pooled and then purified using C18 columns (The Nest Group Inc., Southboro, MA) before running MS.

### Fractionation of iTRAQ-labeled Peptides Using OGE

OGE of peptides was carried out using 24-cm IPG strips, pH 3-10 (GE Healthcare), and an OGE fractionator [17] (Agilent Technologies, Inc.) according to the instructions accompanying the equipment. IEF strip was placed on an OGE tray, gel face up, followed by the placement of the frame. Modified Offgel IEF buffer (150 µl; 4 M urea/10% glycerol/0.5% 3-10 ampholytes) was added per well. Electrode pads were wetted in Offgel IEF buffer and placed at both the ends of the strip. Offgel IEF buffer, 200 µl or 400 µl, was added to the left and right protruding wells, respectively. Prehydration was carried out for 5 h at RT on the leveled Offgel platform. At the end, the residual Offgel IEF buffer was removed by pipetting, and the electrode pads were removed. The tray was laid in a slanted position with the right protruding end at a higher level. The Offgel IEF buffer beneath the strip was removed by adding 600 µl mineral oil to the right protruding end such that the Offgel IEF buffer gravitated to the left end. New electrode pads freshly wetted in Offgel IEF buffer were placed at both the ends. The Offgel IEF buffer was removed from all the wells, and 150 µl of peptide sample prepared in Offgel IEF buffer was added to each well. Mineral oil, 200 µl or 400 µl, was added to the left and right protruding wells, respectively. The cover seal was placed, and the tray was moved to the Offgel fractionator platform. The fractionator was run under the following conditions: 50 kV-h, 8000 V, 50 µA, and 200 W. At the end of the run, the samples were processed as follows. The first five samples (1-5) were collected in one tube. The left electrode pad and right electrode pad were transferred to two separate tubes that contained 100 µl of Offgel IEF buffer. The electrode pads (left and right electrode pad) were processed as follows:

1) Vortex vigorously for 30 s

2) Centrifuge at 21000 rpm for 5 min at RT (Microfuge 22R Centrifuge, rotor F301.5)

3) Recover the solution by squeezing the pad

The extracted solution from the left electrode pad was pooled with OG5 (OGE well 5), and the solution from the right electrode pad was pooled with OG24 (OGE well 24), thus generating a total of 20 fractions. The OGE fractions were purified similar to PIEF fractions using C18 columns prior to MS.

### C18 Spin Column Purification

Peptide fractions resulting from PIEF and OGE were cleaned up before MS runs using Vydac C18 silica microspin columns (The Nest Group, Inc.). Briefly, the column was conditioned with 100 µL of 100% ACN by centrifuging at RT at a low speed about 100 rpm for 1 min (Microfuge 22R Centrifuge, rotor F301.5), followed by flushing out of ACN with 100 µL of H_2_O twice by centrifuging at RT at about 3000 rpm for 1 min. TFA (2%) was added to the sample to a final concentration of 0.1%, and the peptides were allowed to bind to the column by centrifuging at RT at 2000 rpm for 2 min. The bound peptides were washed with 50 µL of 0.1% TFA twice by centrifuging at RT at 2000 rpm for 1 min. Finally, the peptides were recovered using three rounds of elution with 100 µl of 98% ACN, 2% H_2_O and 0.1% formic acid. Our practice of purifying PIEF fractions using C18 spin columns effectively removed any "dust" potentially originating from the filter paper, and the subsequent vacuum drying of samples eliminated any dilution effect (see under Results).

### Cy3 Labeling of Peptides

Cy3 labeling of synthetic peptides as well as complex mixtures of peptides was carried out as described for Cy3 labeling of proteins [35].

### Peptide SDS-PAGE

Conditions were standardized to allow in-lab monitoring of PIEF and OGE to ensure that each procedure proceeded appropriately. Known peptides as well as synthetic peptides with known or predicted pIs were used for this purpose. The peptides tested were as follows: angiotensin (computed pI 6.92 and MW 1296.49), and bradykinin fragment 1-7 (pI 9.75, MW 756.86) (both from Sigma, A9650 and B1651, respectively), and synthetic peptides 72109 and 72120 (both from GenScript, Inc.). Their amino acid sequences, pIs and MWs were as follows: 72109 WK-11: Trp-Val-Gln-Asp-Ser-Met-Asp-His-Leu-Asp-Lys (WVQDSMDHLDK), pI 4.36-4.41, MW 1373.5, and 72120 GK-10: Gly-Tyr-Ser-Ile-Phe-Ser-Tyr-Ala-Thr-Lys (GYSIFSYATK), pI 8.4-8.5, MW 1136.27.

A premade 12% Bis-Tris gel with a 15-well format (Invitrogen) was found to be the best for this task. A lab-made 2× (0.575%) or 4× (1.15%) lithium dodecyl sulfate sample buffer was used, and a 2× buffer consisted of 0.575% lithium dodecyl sulfate (w/v), 20% glycerol and 125 mM Tris, pH adjusted to 6.8 [37]. No dye was included. The samples were prepared such that the final sample solution contained 1× sample buffer and 1× NuPAGE sample reducing agent and were heated at 70°C for 10 min before loading. The gels were run for 30 min using the Mini SureLock system with 500 ul NuPAGE anti-oxidant added to the inner chamber. One well received 1× sample buffer containing tracking dye (but no peptides) to monitor the progression of electrophoresis. The gels were fixed in 40% methanol, 1% glacial acetic acid and 59% distilled/deionized H_2_O for 1-2 h. Gels were stained with Sypro Ruby (Invitrogen) if the peptides used were unlabeled, or imaged using a Typhoon 9410 imager (GE Healthcare) or Molecular Bio-imager (Bio-Rad) if the peptides used were Cy labeled.

### MS

The iTRAQ-labeled and multiplexed peptide samples after fractionation using PIEF and OGE were vacuum-dried and shipped to the University of Victoria Genome BC Proteomics Centre, Victoria, BC, Canada, where LC-MS/MS was carried out on a hybrid Quadrupole-TOF LC-MS/MS Mass Spectrometer (QStar Pulsar i), as in our previous study [25].

### Primary Data Analysis

The objective of the overall project was to determine the utility of PIEF and its relative performance against OGE. Each of the PIEF and OGE datasets was analyzed at the protein level for iTRAQ ratios and at the peptide level for pIs. ProteinPilot Software v2.0 (an updated and integrated version of ProQuant and ProGroup Viewer software applications) and the Paragon method [38] (Applied Biosystems MDS SCIEX) were used to analyze the MS data (.wiff files) to identify and quantify proteins resolved by LC-MS/MS [25]. Bias correction was applied prior to the analysis to correct for differences in total amounts of proteins across samples. The software determines the median average protein ratio and adjusts it to unity and then applies this correction factor to all quantitative results. iTRAQ peptide data were used to search the International Protein Index (Human IPI v. 3.43) protein database. Only proteins that were identified at the >95% confidence level were included in subsequent analysis by exporting the protein summaries as Excel files. Because ProteinPilot was not equipped to provide pI values of the peptides identified, the MS data (.wiff files) were also analyzed using Spectrum Mill Software (Agilent Technologies), aided by QSTAR Data Extractor, and applying the relevant iTRAQ isotope correction factors, and searching the Human IPI protein database (v. 3.43). Peptide summary data, including the pI values, were exported as Excel files using the Excel Export feature of Spectrum Mill.

### Mining Peptide-level pI Data using GeneSpring Software

The use of GeneSpring to mine proteomic data was described earlier [25]. Briefly, the peptide summaries obtained from Spectrum Mill, including columns of information specific for individual peptides, were used to create a custom peptide genome. The columns included: sequence, peptide pI, and others. The custom genome encompassed all the peptides (17,953), identified by PIEF and OGE. Data for each experiment (PIEF and OGE) included columns unique to the experiment, namely file name, and fraction number, as well as sequence, with the sequence column being in common with the custom genome. Thus, sequence was used as a systematic name for analysis using GeneSpring (GX 7.3.1). This allowed us to create lists of peptides (excluding duplicates) detected by both methods as well as the peptides exclusively detected by PIEF or OGE, including the pI values associated with each peptide.

### Mining the Protein-level iTRAQ Data using GeneSpring Software

The IPI protein database, consisting of 72,346 IPI Accession Numbers (representing approximately 24,637 genes) including their gene symbols and other annotations, was used to create a custom protein genome. This required conversion of the IPI database file, first into an Excel file and then into a text file, followed by importing into GeneSpring as a custom genome. Data for each experiment included columns of ratios based on the iTRAQ reagents used 117:114, 117:116, 114:116, and 114:117, as well as gene symbol, with IPI Accession Number being in common with the custom genome. The IPI Accession Number was thus used as the systematic name for GeneSpring analysis purposes. This allowed intersecting of PIEF and OGE protein lists and creating the lists of proteins uniquely detected by each of these methods, as well as the proteins detected by both methods, including their associated iTRAQ ratios. The entire sequence of experimental steps in evaluating the performance of PIEF vs. OGE is outlined in Additional file 4.

### Controlled Testing for the Accuracy of iTRAQ Ratios

To check the reliability of the relative ratios of protein amounts across the four different samples outputted by iTRAQ labeling, a preliminary controlled quantitation study was performed. Known total amounts (25 µg, 75 µg, 125 µg and 175 µg) of a particular protein mixture (consisting of MPC proteins, pI range 5.4-6.2, isolated using a Zoom IEF fractionator) were analyzed using established methods [25,35] (Additional file 4). Briefly, the four protein samples were digested with trypsin, and peptides were differentially labeled with four iTRAQ reagents, 114, 115, 116 and 117, respectively, and multiplexed, followed by desalting using Oasis solid-phase extraction columns and fractionating into 20 fractions according to pI using IPG strip pH 3.0-10.0 and OGE. This study was conducted using only OGE, and PIEF was not performed (due to limitations of laboratory resources and funding). Peptide fractions were purified using C18 spin columns, and eluates containing peptides were divided into two aliquots and subjected to two rounds of reversed-phase LC-MS/MS, with the second run excluding the peptides identified during the first run. Data analysis was performed using ProteinPilot v2.0. Bias correction was not performed because the objective of this particular analysis was to quantify differences purposely introduced into the samples.

## Abbreviations

BLG: beta lactoglobulin; MPC: mesenchymal progenitor cells; OGE: offgel electrophoresis; PIEF: paper IEF; QF: q-proteome fraction; SCX: strong cation exchange; SFC: skin fibroblast cells; USC: unpurified stromal cells

## Competing interests

The authors declare that they have no competing interests.

## Authors' contributions

SB conceived the idea, designed the experiments, analyzed the data, and wrote the manuscript. KR and KC performed the experiments, and participated in discussions. All authors have read and approved the manuscript.

## Supplementary Material

Additional File 1 **Table S1**. Proteins Common to PIEF and OGE. The Supplementary Tables include the MS data and the associated information, such as, Accession Number, Gene Symbol, Gene Description, N (Rank of the specified protein), Unused (ProtScore), Total (ProtScore) and %Cov. *The MS data are provided for each method and differentiated for the proteins detected in common and for those detected by one method only. *Based on the ProteinPilot software user manual, the data attributes may be defined as follows. N represents the rank of the specified protein in relation to the rest of the proteins in the list of detected proteins. Unused (ProtScore) represents a measure of protein confidence for a detected protein calculated using only peptides from spectra that have not already been *used *by higher scoring winning proteins. Notably, once a spectrum is used to identify a protein, it cannot be used to identify any other proteins. The Unused ProtScore is thus the key to how the software identifies proteins. A "good" Unused ProtScore corresponds to the set level of confidence. For 95% confidence, the required Unused ProtScore is 1.3 that is a default in a Paragon Method. Only proteins that show >95% confidence are considered in our study. Total (ProtScore) for a particular protein is calculated using *all *of its peptides and does not necessarily indicate the percent confidence for the identification of a protein. %Cov represents the percentage of the number of amino acids matching to at least one identified peptide divided by the total number of amino acids in the protein sequence. Click here for file

Additional File 2 **Table S2**. Proteins Unique to PIEF Click here for file

Additional File 3 **Table S3**. Proteins Unique to OGE Click here for file

Additional File 4 **Schematic**. Flow diagram outlining the essential steps of sample preparation, cleanup and concentration in comparing the performance of PIEF vs. OGE Click here for file
