# Supplementary material for: Immobilized pH gradient-driven paper-based IEF: a new method for fractionating complex peptide mixtures before MS analysis
Source: Clin Proteomics. 2011 Jun 15;8(1):10. doi: 10.1186/1559-0275-8-10 (PMC3170271; doi:10.1186/1559-0275-8-10)
Supplement: Additional File 4 — Schematic. Flow diagram outlining the essential steps of sample preparation, cleanup and concentration in comparing the performance of PIEF vs. OGE [file 1559-0275-8-10-S4.DOCX]

**Schematic 1: Flow diagram outlining the essential steps of sample preparation, cleanup and concentration in comparing the performance of PIEF vs. OGE**

Cell culture and protein extraction

Acetone precipitation of proteins

Resolubilization and protein estimation

Reduction and alkylation of proteins

Desalting followed by acetone precipitation of 110-µg aliquots

Trypsin digestion followed by drying

iTRAQ labeling followed by multiplexing and drying

OASIS desalting followed by drying

Division of sample equally between PIEF and OGE

Carrying out PIEF and OGE

Recovery of peptides from IEF fractions and C18 column purification

MS runs and subsequent data analysis of PIEF and OGE fractions,

using identical methods and database searches
